# Rapamycin Controls Lymphoproliferation and Reverses T-Cell Responses in a Patient with a Novel *STIM1* Loss-of-Function Deletion

**DOI:** 10.1007/s10875-024-01682-0

**Published:** 2024-04-05

**Authors:** Ibrahim Serhat Karakus, Mehmet Cihangir Catak, Alexandra Frohne, Feyza Bayram Catak, Melek Yorgun Altunbas, Royala Babayeva, Sevgi Kostel Bal, Sevgi Bilgic Eltan, Ezgi Yalcin Gungoren, Fehim Esen, Itir Ebru Zemheri, Elif Karakoc-Aydiner, Ahmet Ozen, Suar Caki-Kilic, Michael J. Kraakman, Kaan Boztug, Safa Baris

**Affiliations:** 1 School of Medicine, Marmara University, Istanbul, Turkey; 2 Division of Pediatric Allergy and Immunology, School of Medicine, Marmara University, Fevzi Çakmak Mah. No: 41, Pendik/Istanbul, Turkey; 3 Istanbul Jeffrey Modell Diagnostic and Research Center for Primary Immunodeficiencies, Istanbul, Turkey; 4 The Isil Berat Barlan Center for Translational Medicine, Istanbul, Turkey; 5 Anna Children’s Cancer Research Institute, Vienna, Austria; 6 Department of Ophthalmology, School of Medicine, Istanbul Medeniyet University, Istanbul, Turkey; 7 Department of Pathology, Umraniye Education and Research Hospital, University of Health Sciences, Istanbul, Turkey; 8 Division of Pediatric Hematology, Umraniye Education and Research Hospital, University of Health Sciences, Istanbul, Turkey; 9 Department of Pediatrics and Adolescent Medicine, Medical University of Vienna, Vienna, Austria; 10 CeMM Research Center for Molecular Medicine of the Austrian Academy of Sciences, Vienna, Austria; 11 Anna Children’s Hospital, Vienna, Austria

**Keywords:** Stromal interaction molecule 1, Ca^2+^ release-activated calcium channel, circulating T follicular helper cells, regulatory T cells, rapamycin

## Abstract

**Purpose:**

Deficiency of stromal interaction molecule 1 (STIM1) results in combined immunodeficiency accompanied by extra-immunological findings like enamel defects and myopathy. We here studied a patient with a STIM1 loss-of-function mutation who presented with severe lymphoproliferation. We sought to explore the efficacy of the mTOR inhibitor rapamycin in controlling disease manifestations and reversing aberrant T-cell subsets and functions, which has never been used previously in this disorder.

**Methods:**

Clinical findings of the patient were collected over time. We performed immunological evaluations before and after initiation of rapamycin treatment, including detailed lymphocyte subset analyses, alterations in frequencies of circulating T follicular helper (cT_FH_) and regulatory T (Treg) cells and their subtypes as well as T cell activation and proliferation capacities.

**Results:**

A novel homozygous exon 2 deletion in *STIM1* was detected in a 3-year-old girl with severe lymphoproliferation, recurrent infections, myopathy, iris hypoplasia, and enamel hypoplasia. Lymphoproliferation was associated with severe T-cell infiltrates. The deletion resulted in a complete loss of protein expression, associated with a lack of store-operated calcium entry response, defective T-cell activation, proliferation, and cytokine production. Interestingly, patient blood contained fewer cT_FH_ and increased circulating follicular regulatory (cT_FR_) cells. Abnormal skewing towards T_H_2-like responses in certain T-cell subpopulations like cT_FH_, non-cT_FH_ memory T-helper, and Treg cells was associated with increased eosinophil numbers and serum IgE levels. Treatment with rapamycin controlled lymphoproliferation, improved T-cell activation and proliferation capacities, reversed T-cell responses, and repressed high IgE levels and eosinophilia.

**Conclusions:**

This study enhances our understanding of STIM1 deficiency by uncovering additional abnormal T-cell responses, and reveals for the first time the potential therapeutic utility of rapamycin for this disorder.

**Supplementary Information:**

The online version contains supplementary material available at 10.1007/s10875-024-01682-0.

## Introduction

Calcium (Ca^2+^) homeostasis plays a pivotal role in lymphocyte activation, maturation, and signal transduction [1]. Calcium entry into the immune cells is enabled through Ca^2+^ release-activated calcium (CRAC) channels, which contain 2 different proteins named calcium release-activated calcium modulator (ORAI) and stromal interaction molecule (STIM). STIM1 and STIM2 proteins function as calcium sensors within the endoplasmic reticulum (ER). As ER calcium levels become depleted during cell activation, STIM1 and STIM2 undergo conformational changes. These enable them to bind to ORAI1, ORAI2, and ORAI3 proteins, forming a functional CRAC channel in the plasma membrane. The opening of the CRAC channel facilitates a sustained influx of Ca^2+^ from the extracellular space. This mechanism is called store-operated Ca^2+^ entry (SOCE), and it is essential not only for effective immune system functioning but also for platelet activation, muscle contraction, and osteoblastogenesis [2–4]. Human biallelic loss-of-function (LOF) mutations in *ORAI1* and *STIM1* disrupt Ca^2+^ influx, causing a syndrome called CRAC channelopathy characterized by immunodeficiency, autoimmunity and other non-immunological findings [5, 6].

Previously, LOF or gain-of-function (GOF) mutations were described in the *STIM1* gene. GOF mutations are associated with autosomal dominant Tubular Aggregate Myopathy and Stormorken syndrome [7, 8]. In contrast, LOF mutations affecting STIM1 results in a combined immunodeficiency (CID) accompanied by autoimmunity, ectodermal dysplasia presented as dental enamel defects and anhydrosis, and non-progressive myopathy characterized by muscular hypotonia and partial iris hypoplasia causing mydriasis [4, 9, 10]. In 2009, Picard et al. described the first three patients with a homozygous recessive nonsense mutation in the *STIM1* gene, leading to the formation of a truncated protein [5]. After that, 14 additional patients were reported in the literature [5, 11–19]. In general, STIM1-deficient patients present within the first year of life, and CID phenotype requires hematopoietic stem cell transplantation (HSCT) [9]. Developmental delay, recurrent broad infections, immune dysregulation, lymphoproliferation, anhydrosis, mydriasis, iris hypoplasia, and myopathy are the most commonly encountered findings in those patients. However, the clinical presentations are highly variable, encompassing a spectrum from life-treating manifestations [5, 12] to diseases without immune deficiency or myopathy [13, 15]. Patients with STIM1 deficiency usually have normal frequencies of T, B, and natural killer (NK) cells, and the T cells show a broad repertoire [12, 16]. However, due to insufficient Ca^2+^ influx, defective T-cell and NK-cell function is a hallmark phenomenon in most patients.

Here, we describe a 3-year-old girl with a novel homozygous exon 2 deletion in *STIM1* who presented with recurrent pneumonia, multiple lymphadenopathies, enamel hypoplasia, and axial hypotonia. Perplexingly, the most prominent clinical feature of the patient was the widespread excessive large lymph nodes. This atypical presentation was deceptive and misdiagnosed as an autoimmune lymphoproliferative syndrome (ALPS). Her lymphoproliferation responded very well to rapamycin, which to our knownledge has not been used previously to treat STIM1-deficient patients. We observed that treatment with rapamycin resulted in the restoration of naïve T cells and circulating follicular helper T (cT_FH_) cells, along with their subtypes. Additionally, we noted an increase in T-cell activation and proliferative capacity. Our report expands the clinical spectrum of the disease and provides new insights into the treatment.

## Materials and Methods

### Clinical Assessments

The local ethics committee from Marmara University approved the clinical and research studies protocol, and written informed consent was obtained from the family. We documented the clinical and demographic features of the patient and provided data regarding long-term follow-up.

### Cell Culture

T lymphoblasts were generated from the ex vivo expansion of peripheral blood mononuclear cells (PBMCs) with Phytohemagglutinin (PHA, 1 μg/mL) and IL-2 (100 U/mL) in RPMI-1640 media containing 5% human serum, HEPES, Penicillin–Streptomycin, non-essential amino acids and sodium pyruvate and co-cultured with irradiated feeder cells (PBMCs from healthy donors) in a ratio of 1:1. T lymphoblasts were split every 2–3 days with fresh IL-2 (50 U/mL) added. T lymphoblasts were used after 9–13 days of expansion. EBV-transformed B cell lines were generated by incubating PBMCs (~ 5 × 10^6^ cells) with EBV-containing supernatant at a volume ratio of 1:1 in RMPI-1640 media supplemented with 10% FCS, 1% Penicillin–Streptomycin, and 1% HEPES. After at least 2 h, cyclosporine A (5 μg/mL) was added to inhibit T cells before prolonged cultivation until the EBV-immortalized B cells were established. Subsequent experiments using these cell lines were performed without prior treatments or stimulations other than those required for their expansion and maintenance.

### Antibodies and Flow Cytometry

Peripheral blood lymphocyte subset analyses, intracellular cytokine staining, upregulation, and proliferation assays were performed by flow cytometry, as described previously [20–25]. Age-matched healthy donors were used for comparisons. Stained cells were acquired by Navios EX cytometer (Beckman Coulter) and analyzed by FlowJo software (TreeStar, Ashland, Ore). The details are presented in the Supplementary file.

### Store-Operated Calcium Entry (SOCE) Assay

T lymphoblasts (1 × 10^6^) were washed in Ca^2+^-free PBS and stained with Calcium Sensor Dye eFluor™ 514 (eBiosciences) for 30 min at 37℃. Cells were washed and resuspended in PBS containing 1 mM EGTA. Cells were first incubated with 1 μg/mL anti-CD3 (OKT3, eBioscience) for 5 min at 37C before a 30 s baseline measurement was recorded. Then, 20 μg/mL of AffiniPure goat anti-Mouse IgG (Jackson ImmunoResearch) was added to induce T-cell receptor (TCR) crosslinking, followed by 3 min of measurement. Finally, CaCl (2 mM final concentration) to induce SOCE was added, and a further 2 min was recorded. The assay was performed using an LSRFortessa™ (BD) and analyzed using FlowJo (Ver. 10). Results, recorded as intensity per unit time, were normalized to the mean basal intensity and plotted against time.

### Western Blotting

T- lymphoblasts and EBV-transformed B-cells were washed in PBS, pelleted by centrifugation, and lysed in Frackeltons buffer containing protease and phosphatase inhibitors. Lysate protein concentration was determined using a DC reagent (Bio-Rad). Samples (25 μg) in Laemmli Buffer were separated on acrylamide gels (10%) at 75 V for 45 min followed by 120 V and transferred to PVDF membranes at 110 V for 90 min, or 40 V overnight at 4℃. Membranes were washed in TBST, blocked in 5% milk, and incubated in primary antibody overnight at 4℃. The following primary (STIM1—1:1000, Cell Signaling Technology, #5668; β-actin—1:5000, Sigma Aldrich, #A1978; HSP90α/β—1:5000, Santa Cruz Biotechnology, #sc-13119) and secondary (anti-Mouse HRP—BD Biosciences, #554002; anti-Rabbit HRP—Bio-Rad, #172–1019) antibodies were used.

### IL-2 Production Assay

Intracellular cytokine production was assessed in T-lymphoblasts by stimulating 0.2 × 10^6^ cells for 5 h with Phorbol 12-myristate 13-acetate (PMA, 0.2 mM) and Ionomycin (1 μg/mL). Brefeldin A was added during the final 4 h of the stimulation. Cells were washed and stained for T-cell surface markers (CD4, CD8) on ice for 30 min. Subsequently, cells were fixed, permeabilized, and stained with IL2. An LSRFortessa™ (BD) was used for acquisition, and plots were analyzed using FlowJo (Ver. 10).

### Whole-Exome Sequencing

The genetic analysis was performed using whole-exome sequencing (WES). Briefly, genomic DNA was extracted from peripheral blood samples, and 1 μg of DNA was used for library preparation using the Twist Exome 2.0 kit. Paired-end (100 bp) sequencing was performed on an Illumina NovaSeq 6000 device. The homozygous *STIM1* deletion was called with the ExomeDepth software [26], validated by long-range PCR followed by Sanger sequencing, and segregated in all available family members.

## Results

### A novel STIM1 Mutation (STIM1^Ex2del^) in a Patient with Severe Lymphoproliferation

**Fig. 1:**
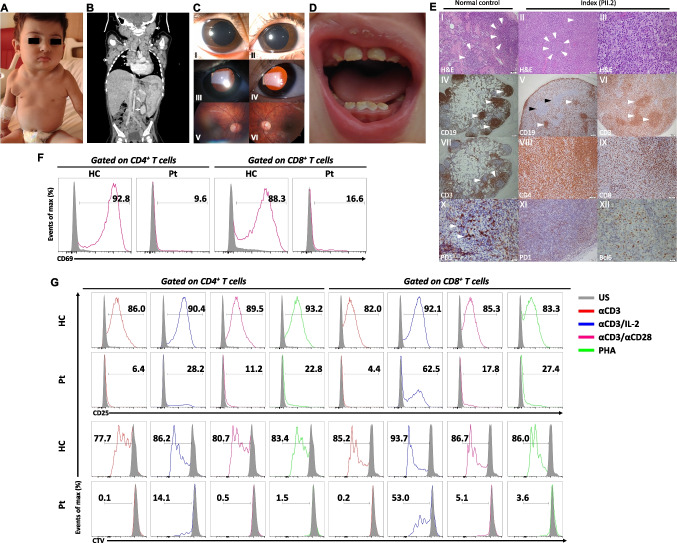
Clinical and immunological phenotype of STIM1^Ex2del^ patient. (**A**) Profound lymph node enlargements in the cervical and axillary areas. (**B**) The CT scan reveals hepatosplenomegaly and conglomerated lymph node enlargements in the mediastinal, abdominal, cervical, and axillary areas indicated by the white arrows. (**C**) Slit lamp examination of the anterior segment revealed pupillary dilation and iris stroma hypoplasia, accompanied by the absence of iris crypts **(I** and **II)**. Retro-illumination of the eye unveiled atrophic, thin, band-like iris structures over the lens, reminiscent of persistent pupillary membranes **(III** and **IV)**. The pupil was non-responsive to light. Fundus examination was unremarkable, except for slight optic disc pallor **(V** and **VI)**. (**D) **A dental examination indicates normal-sized teeth with brown and creamy-colored enema and dental attrition. (**E**) Lymph node biopsy results: **(I, H&E)** Normal follicles (arrows). **(II, H&E)** Small regressed lymphoid follicles (arrows) and T zone expansion eliminating the normal structure. **(III, H&E)** In T zone, atypical proliferation constitutes medium-sized cells with nucleolies adjacent to the nuclear membrane, large-sized cells with central single nucleole, histiocytes, and small lymphoid cells. Immunohistochemical staining showing **(IV)** normal B cells localization (arrows). **(V)** CD20 positivity in regressed and damaged lymphoid follicles (white arrows) and abnormal large cells in the T zone (black arrow). **(VI)** T lymphoid cells stained with CD3 in diffuse and nodal pattern. **(VII)** Normal T cells localization (arrows). **(VIII)** Diffuse staining of CD4^+^ T cells. **(IX)** Scattered CD8^+^ T cells. **(X)** Normal germinal center positivity with PD1. **(XI-XII)** Sparse staining of PD1 and Bcl6 positivity in some CD4^+^ T cells. Original magnifications: IV, V, VI, VII: 4x; I, II, VIII, IX, XI: 20x; III, X, XII: 40x. (**F**) Representative flow cytometric analysis of percentages of CD69 in CD4^+^ and CD8^+^ T cells in the patient and healthy control with unstimulated condition and stimulated (αCD3/CD28, 1 μg/mL each) condition for 24 h. (**G**) Representative flow cytometric analysis of percentages of CD25 and proliferation in CD4^+^ and CD8^+^ T cells in the patient and healthy control with unstimulated condition (grey) and stimulated (αCD3 (1 μg/mL, red line), αCD3/IL-2 (1 μg/mL and 100 U/mL, blue line), αCD3/CD28 (1 μg/mL each, purple line), and PHA (10 μg/mL, green line)) conditions for 72 h. H&E: hematoxylin and Eosin, Pt: patient, HC: healthy control, US: unstimulated, αCD3: anti-CD3, αCD3/CD28: anti-CD3/CD28, PHA: phytohemagglutinin, CTV: cellTrace Violet

A 3-year-old girl (PII.2), born to consanguineous parents, was admitted to our clinic at 16 months of age with bilateral axillary lumps and occasional episodes of fever. She was born prematurely and had esophageal atresia. She underwent repair surgery and stayed in the intensive care unit for one month. Her medical history was remarkable for recurrent hospitalizations due to pneumonia with high erythrocyte sedimentation rates (range: 74-120 mm/hr) and C-reactive protein levels (range: 6-47 mg/L). She also had anhidrosis with some suspected associated fevers. She also experienced an impetigo-like infectious cutaneous lesion due to *Staphylococcus aureus* infection. She received standard childhood vaccinations without any complications. Upon physical examination, the liver and spleen were palpable, 3 cm and 2 cm below the edge of the ribs, respectively. Additionally, multiple enlarged lymph node conglomerates were detected in the cervical, supraclavicular, axillary, mediastinal, abdominal, and inguinal regions (Fig. 1A and B). Her skin exhibited xerosis and pruritus without eczema. She had a kyphotic posture with mild axial hypotonia. Ophthalmologic examination showed mydriasis, iris hypoplasia, and persistent pupillary membranes over the lens (Fig. 1C). The pupils were non-responsive to light. Additionally, her teeth had brownish enamel with hypoplasia and increased notches (Fig. 1D).

Laboratory results revealed severe leukocytosis, eosinophilia, and elevated serum IgG levels (Table 1). She had adequate IgG antibodies against protein antigens and sufficient isohemagglutinin responses. She also had significantly high serum total IgE level and specific IgE to egg white without clinical symptoms upon exposure to egg. Her creatinine kinase level was in a normal range. The direct Coombs test was positive in the sera without signs of autoimmune cytopenia. At the same time, we did not detect positivity for other rheumatological autoantibodies, except for antinuclear antibody (1/320). Blood Epstein-Barr virus (EBV) and cytomegalovirus (CMV) PCR were negative. We performed bone marrow and axillary lymph node biopsies to exclude hematological and lymphoid malignancies. While the bone marrow examination was normal, axillary lymph node biopsy revealed atypical lymphoid proliferation. In the lymph node, pronounced T zone hyperplasia was identified, resulting in the regression of lymphoid follicles and disruption of the characteristic nodal architecture. Regressed follicles, as evidenced by CD20 and CD23 staining, were observed. Notably, the T cells exhibited diffuse positivity for CD4, while CD8 positivity was scattered. There was very sparse staining in CD4^+^ T cells with PD1 and Bcl6, delineating reduced resident T_FH_ cells in follicular centers (Fig. 1E). Additionally, kappa and lambda-positive polyclonal plasma cells were present, predominantly localized beneath the capsule. The biopsy material was negative for HHV-8, EBV, CMV, *Mycobacterium tuberculosis,* and *Bartonella henselae*. Due to the severe lymphoproliferation without malignancy, we considered a probability of ALPS-like disease.

**Table 1:** Laboratory evaluation of the STIM1-deficient patient

Parameters	Before rapamycin (age: 16 mos)	On rapamycin (at 6 months of therapy)	On rapamycin (at 20 months of therapy)	Reference values (1–2 years)	Reference values (2–5 years)
Leukocyte count (/mm^3^)	38,200 (↑)	14,600	7070	6000–17500	5200–13000
Absolute lymphocyte count	21,200 (↑)	11,200	4800	2963–11,346	2175–7578
Absolute neutrophil count	5240	2200	1610	1192–9147	1261–7576
Absolute eosinophil count	8900 (↑)	350 (↑)	210	< 500	< 500
Creatine kinase	33	-	-	< 250	< 250
IgG (mg/dl)	4500 (↑)	1057 (on IVIG)	3062 (↑) (on IVIG)	605–1430	604–1941
IgA (mg/dl)	17 (↓)	19 (↓)	19 (↓)	30–307	26–296
IgM (mg/dl)	198	46 (↓)	142	66–228	71–235
IgE (IU/ml)	6500 (↑)	180 (↑)	25	< 50	< 50
Specific antibody titers					
Anti Hbs IgG (mIU/ml)	84	-	-	0–10	0–10
Anti-measles IgG (U/ml)	548	-	-	> 200	> 200
Anti-mumps IgG (U/ml)	96.5	-	-	> 100	> 100
Anti-varicella zoster IgG (mIU/ml)	280	-	-	> 100	> 100
Isohemagglutinin (Anti-B) level	1/8	-	-	≥ 1/8	≥ 1/8
Lymphocyte subsets					
CD3^+^ T cells, (%)	74.4	81.4	80.8	52–84	57–83
CD3^+^ count (/mm^3^)	15,688 (↑)	9116 (↑)	3878	1982–7372	1432–5445
CD3^+^ CD4^+^, (%)	44.3	64.9 (↑)	62.7 (↑)	29–58	25–55
CD3^+^ CD4^+^ count (/mm^3^)	9391 (↑)	7268 (↑)	3009	1211–4696	722–3202
CD3^+^ CD8^+^, (%)	30	15.8	16.9	14–31	14–39
CD3^+^ CD8^+^ count (/mm^3^)	6360 (↑)	1769	811	567–2494	387–2303
CD19^+^ B cells, (%)	8.2 (↓)	15.7	14.6	13–37	10–31
CD19^+^ count (/mm^3^)	1738	1758	700	526–3126	322–1633
CD16^+^56^+^ NK cells, (%)	6.6	1.4 (↓)	1.9 (↓)	2–26	2.5–29
CD16^+^56^+^ NK cell count (/mm^3^)	1399	156	91.2	105–1461	88–1391
CD19^+^CD27^−^IgD^+^ B cells, (%)	64.3 (↓)	85.1	82.8	69–97	55–95
CD19^+^CD27^+^IgD^+^ B cells, (%)	6.1	7.2	7.6	4–19	6–23
CD19^+^CD27^+^IgD^−^ B cells, (%)	14.8	4.2	4.1	2–17	3–32
CD21^low^ CD38^low^ activated B, (%)	21.7 (↑)	8.5	8.5	0.9–9	0.9–11
CD3^+^ TCR^α/β^ cells, (%)	93.3	94.7	95.5	87–98	79–100
CD3^+^ TCR^γ/δ^ cells, (%)	3.9	3.1	3	2–14	3–27
CD4^+^ CD45RA^+^ CD31^+^ T cells, (%)	16.2 (↓)	19.8 (↓)	25.8 (↓)	60–83	49–78
CD4^+^ CD45RA^+^ CCR7^+^ T cells, (%)	16.5 (↓)	22.7 (↓)	27.5 (↓)	52–97	49–90
CD4 ^+^ CD45RA^−^ CCR7^+^ T cells, (%)	10.6	9.6	9.4 (↓)	8–38	13–44
CD4^+^ CD45RA^−^ CCR7^−^ T cells, (%)	63.1 (↑)	54.7 (↑)	57.5 (↑)	0.3–11	0.8–10
CD4^+^ CD45RA^+^ CCR7^−^ T cells, (%)	9.7	12.8	5.4	0.2–55	0.1–58
CD8^+^ CD45RA^+^ CCR7^+^ T cells, (%)	12.4 (↓)	46.4	45.1	26–90	21–100
CD8^+^ CD45RA^−^ CCR7^+^ T cells, (%)	3.8	3	1.6	0.9–8	0.9–9
CD8^+^ CD45RA^−^ CCR7^−^ T cells, (%)	40.5 (↑)	17.7	27.7	3–40	5–32
CD8^+^ CD45RA^+^ CCR7^−^ T cells, (%)	43.2	32.8	25.3	13–69	13–63

Abbreviations: *IVIG* Intravenous immunoglobulin, *Mos* Months, naive mature B cells (CD19^+^CD27^–^IgD^+^), non-class-switched memory B cells (CD19^+^CD27^+^IgD^+^), class-switched memory B cells (CD19^+^CD27^+^IgD^-^), autoreactive B cells (CD19^+^CD21^low^CD38^low^), recent thymic emigrants (CD4^+^CD45RA^+^CD31^+^), CD4^+^ naïve T cells (CD4^+^CD45RA^+^CCR7^+^), central memory CD4^+^ T cells (CD4^+^CD45RA^–^CCR7^+^), effector memory CD4^+^ T cells (CD4^+^CD45RA^–^CCR7^-^), terminally differentiated effector memory CD4^+^ T cells (CD4^+^CD45RA^+^CCR7^–^), CD8^+^ naive T cells (CD8^+^CD45RA^+^CCR7^+^), central memory CD8^+^ T cells (CD8^+^CD45RA^–^CCR7^+^), effector memory CD8^+^ T cells (CD8^+^CD45RA^–^CCR7^–^), terminally differentiated effector memory CD8^+^ T cells (CD8^+^CD45RA^+^CCR7^–^), Abnormal values are indicated in the parenthesis with arrows

The immunological evaluation was notable for CD3^+^ T lymphocytosis with skewing of CD4^+^ and CD8^+^ T cells towards a memory phenotype. In addition, recent thymic emigrant cells were decreased compared with the healthy control. Increased CD21^low^ CD38^low^ activated B cells were also detected (Table 1). The immune system was further evaluated by lymphocyte proliferation, CD69 and CD25 activation. The patient showed blunted CD69 expression after stimulation with αCD3/CD28 compared with healthy control (Fig. 1F). Furthermore, the patient’s CD4^+^ and CD8^+^ T cells exhibited drastically impaired CD25 expression and proliferation following stimulation with αCD3, αCD3/CD28, and PHA compared with healthy controls. The T cells showed higher proliferation and activation after αCD3/IL-2 stimulation but still lower than controls (Fig. 1G). Based on these results, at 17 months, the patient was commenced on prophylactic antibiotic therapy and intravenous immunoglobulin replacement, which resulted in the control of infections. Her acute phase reactants were also effectively normalized, suggesting the absence of non-infectious inflammatory processes contributing to elevated levels.

### Identification of a Pathogenic *STIM1* Variant

**Fig. 2:**
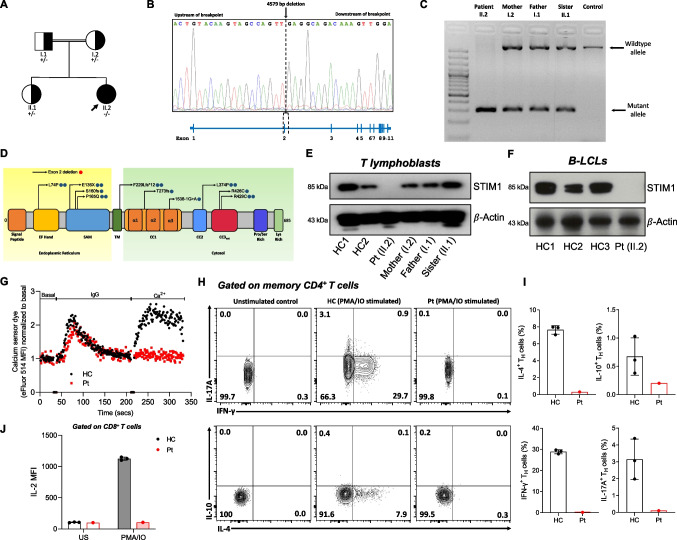
Identification of a STIM1^Ex2del^ deletion in a child with severe lymphoproliferation. (**A**) Pedigree of the patient with STIM1^Ex2del^ variant. Double lines indicate consanguinity; a filled black circle depicts the patient; half-filled black circles or squares depict the carriers. Squares and circles distinguish males and females, respectively. (**B**) Sanger sequencing analysis of the *STIM1* 11:3967441_3972019del (hg38) deletion in the index patient with schematic presentation of the location of the deleted sequence within the *STIM1* gene. (**C**) Gel showing long-range PCR products of the patient (homozygous for the 4579 bp deletion), the heterozygous family members, and an unrelated control (homozygous wild type). (**D**) Schematic diagram of STIM1 protein domains, with locations of known (blue) and novel (red) mutations. Amino acid changes are presented by their single-letter code. SAM: sterile-α motif, TM: transmembrane domain, CC: cytosolic coiled coil. (**E**) Western blot analysis of the STIM1 protein in the patient, mother, father, sister, and healthy controls in T lymphoblasts and (**F**) EBV immortalized B-lymphoblastoid cell lines (LCL). (**G**) SOCE assay in T lymphoblasts from the patient and a healthy control. (**H**) Flow cytometric analysis with representative plots of IL-4, IL-17A, IFN-γ, and IL-10–producing circulating memory CD4^+^ T cells after PMA (50 ng/mL) and ionomycin (1 μg/mL) stimulation for 6 h. (**I**) The percentages of the IL-4^+^, IL-17A^+^, IFN-γ^+^, and IL-10^+^ T_H_ cells in the patient and healthy controls. (**J**) Mean fluorescence intensity of IL-2 in the patient and healthy controls. Pt: patient, HC: healthy controls, MFI: mean fluorescence intensity, PMA/IO: phorbol myristate acetate and ionomycin

The WES and copy number variation analysis revealed a novel homozygous deletion in the *STIM1* gene. The deletion (11:3967441_3972019del, hg38) comprises 4579 bp, including exon 2 and parts of its flanking introns (NM_001382567.1). This deletion was confirmed by Sanger sequencing (Fig. 2A and B). The parents and healthy sister were carriers for the genetic deletion, as confirmed by variant segregation using long-range PCR (Fig. 2C). This novel mutation and previously reported mutations are depicted in Fig. 2D. The list of other rare mutations detected in the patient is presented in Table S1. When variants with high combined annotation dependent depletion scores, identified by WES, were examined in detail, they did not explain the patient's ALPS-like phenotype and accompanying esophageal atresia findings. However, some variants with functions not fully explored may still contribute to the clinical presentation.

The STIM1^Ex2del^ variant caused a total abolishment of STIM1 protein expression in the patient’s derived T lymphoblasts and EBV-cell lines (Fig. 2E and F). Consistent with the identified STIM1 defect, the SOCE assay showed a complete loss of extracellular Ca^2+^ uptake following ER calcium depletion compared with the healthy control (Fig. 2G), as in previously reported patients [5, 12]. We also measured cytokine expression in isolated peripheral blood mononuclear cells by gating on CD4^+^CD45RO^+^ memory T cells after stimulation with PMA and ionomycin in vitro. The production of IFN-γ, IL-4, IL-10, and IL-17A was strongly reduced in the patient compared with healthy controls (Fig. 2H and I). Furthermore, stimulation-induced IL-2 expression was absent in T lymphoblast cells (Fig. 2J). Overall, these analyses strongly suggest that the STIM1^Ex2del^ variant is deleterious and acts as a LOF mutation, leading to impaired activation, proliferation, and cytokine production of T cells.

### The STIM1^Ex2del^ Deletion is Associated with Lymphoproliferation and Dysregulated T-Cell Responses

**Fig. 3:**
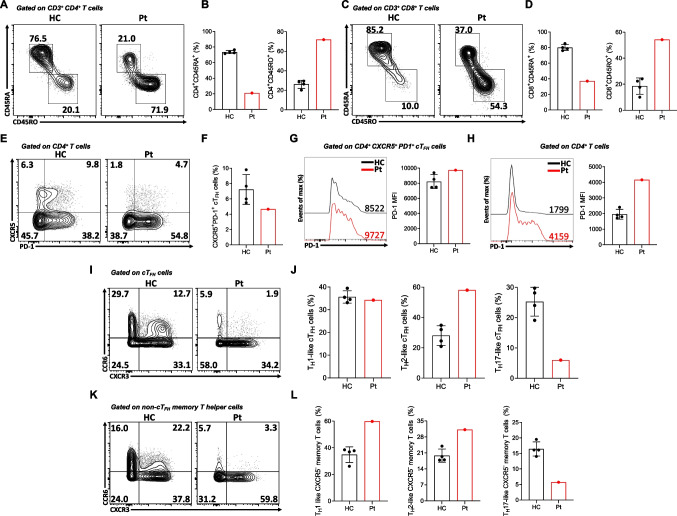
Reduced cT_FH_ cells and skewed T_H_2-like responses in STIM1^Ex2del^ deficiency. Representative plots (**A**) and percentages (**B**) of naïve (CD3^+^CD4^+^CD45RA^+^CD45RO^−^) and memory (CD3^+^CD4^+^CD45RO^+^CD45RA^−^) CD4^+^ T cells in the patient and healthy controls. Representative plots (**C**) and percentages (**D**) of naïve (CD3^+^CD8^+^CD45RA^+^CD45RO^–^) and memory CD3^+^CD8^+^CD45RO^+^CD45RA^–^) CD8^+^ T cells in the patient and healthy controls. Representative plots (**E**) and percentages (**F**) of cT_FH_ cells of the patient and healthy controls. Representative histogram of PD-1 expression in cT_FH_ (**G**) and total CD4^+^ T **(H)** and cells with mean fluorescence intensity of PD-1 in the patient and healthy controls. (**I**) Representative plots of subtypes of the cT_FH_ cells. (**J**) The percentages of subtypes of the cT_FH_ cells compared to healthy controls. (**K**) Representative plots of subtypes of the non-cT_FH_ memory T cells. **(L)** The percentages of subtypes of the non-cT_FH_ memory T cells compared to healthy controls. Pt: patient, HC: healthy controls, MFI: mean fluorescence intensity, cT_FH_: circulating T follicular helper cell, PD-1: programmed cell death protein 1

Mice deficient in both Stim1 and Stim2 in T cells develop a lymphoproliferative disorder and dermatitis, characterized by eosinophilia and augmented IgE and IgG1 responses. Furthermore, these mice display diminished percentages of regulatory T (Treg) and follicular regulatory T (T_FR_) cells with increased numbers of T_FH_ in splenocytes, underscoring the role of STIM1/STIM2 in controlling T cell responses [27, 28]. Interestingly, the development of T_FH_ cells in mice with T cell-specific deletion of Stim1/2 was severely impaired after acute viral infection or immunization [29]. Based on these findings in mice, we investigated for the first time the frequencies of circulating T-helper cell subtypes in human STIM1 deficiency. These included cT_FH_ (CD4^+^CXCR5^+^CD45RA^–^ and CD4^+^CXCR5^+^PD1^+^), non-cT_FH_ memory T-helper cells (CD4^+^CXCR5^–^CD45RA^–^), Treg (CD4^+^CD25^hi^CD127^lo^) and circulating (c)T_FR_ (CD4^+^CXCR5^+^CD45RA^–^CD25^hi^CD127^lo^) cells. The gating strategy of these analysis are presented in Fig.S1 and Fig.S2. The patient with the STIM1^Ex2del^ variant exhibited an increased frequency of circulating memory CD4^+^/CD8^+^ T cells (CD3^+^CD4^+^/CD8^+^CD45RA^–^CD45RO^+^) and a decreased frequency of circulating naïve CD4^+^/CD8^+^ T cells (CD3^+^CD4^+^/CD8^+^CD45RA^+^CD45RO^–^) (Fig. 3A-D). A more detailed analysis of the CD4^+^ T cells compartment showed that the patient had a decreased percentage of cT_FH_ cells with slightly increased PD-1 expression (Fig. 3E-G). The increased PD-1 expression was more prominent in total CD4^+^ T cells, indicating their activation (Fig. 3H). Further, when we evaluated the subtypes of cT_FH_, they displayed a higher percentage of the T_H_2-cell-like phenotype (CD4^+^CXCR5^+^CD45RA^–^CD25^lo^CD127^hi^CXCR3^–^CCR6^–^) compared with healthy controls and a trend for fewer of the T_H_17-cell-like (CD4^+^CXCR5^+^CD45RA^–^CD25^lo^CD127^hi^CXCR3^–^CCR6^+^) phenotype. The T_H_1-cell-like (CD4^+^CXCR5^+^CD45RA^–^CD25^lo^CD127^hi^CXCR3^+^CCR6^–^) percentage was comparable with healthy controls (Fig. 3I and J). On the other hand, patient non-cT_FH_ memory T-helper effector cells exhibited greater skewing towards T_H_1- and T_H_2-cell-like phenotypes (Fig. 3K and L).

**Fig. 4:**
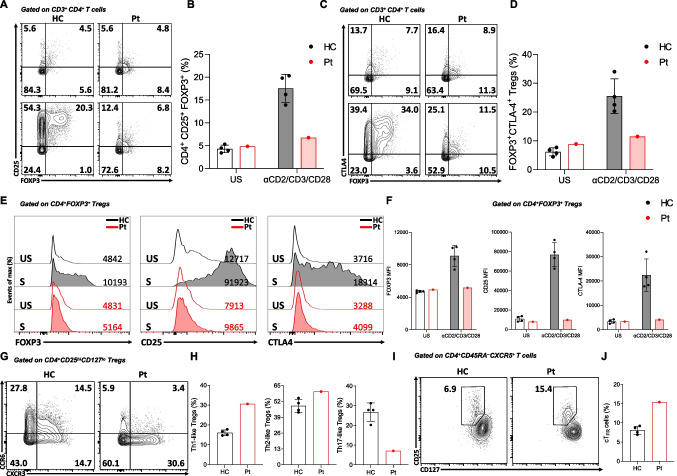
Impaired FOXP3, CD25, and CTLA-4 expression in STIM1^Ex2del^ T_reg_ and skewing towards T_H_1 and T_H_2 responses. Representative plots (**A**) and percentages (**B**) of CD4^+^ CD25^+^FOXP3^+^ Tregs in the patient and healthy controls with unstimulated condition (upper panel) and stimulated condition with αCD2/CD3/CD28-coated beads (1 bead to 2 cells) for 16 h (bottom panel). Representative plots (**C**) and percentages (**D**) of FOXP3^+^CTLA-4^+^ Tregs in the patient and healthy controls with unstimulated condition (upper panel) and stimulated condition with αCD2/CD3/CD28-coated beads (1 bead to 2 cells) for 16 h (bottom panel). (**E**) Representative histograms of FOXP3, CD25, and CTLA-4 expressions in the patient and healthy controls gated on CD4^+^FOXP3^+^ Tregs with unstimulated and stimulated (αCD2/CD3/CD28) conditions (Black line: healthy control unstimulated, Grey filled: healthy control stimulated, Red line: patient unstimulated, Red filled: patient stimulated). (**F**) Mean fluorescence intensity of FOXP3, CD25, and CTLA-4 expressions in patient’s and healthy controls’ CD4^+^FOXP3^+^ Tregs with unstimulated condition and stimulated condition with αCD2/CD3/CD28-coated beads (1 bead to 2 cells) for 16 h. Representative plots (**G**) and percentages (**H**) of subtypes of Treg cells in the patient compared to healthy controls. Representative plots (**I**) and percentages (**J**) of cT_FR_ cells in the patient and healthy controls. Pt: patients, HC: healthy controls, Treg: regulatory T cells, cT_FR_: circulating T follicular regulatory cell

Treg cell frequencies were normal at baseline; however, this cell population had reduced expression of canonical markers, including CD25, FOXP3, and CTLA4, especially after stimulation (Fig. 4A-F). Like other T-helper cell phenotypes, expanded percentages of T_H_1-(CD4^+^CD25^hi^CD127^lo^CXCR3^+^CCR6^–^) and T_H_2-like (CD4^+^CD25^hi^CD127^lo^CXCR3^–^CCR6^–^) Treg cells were observed in the patient, indicating their abnormal reprogramming. In contrast, percentages of T_H_17-cell-like Treg cells (CD4^+^CD25^hi^CD127^lo^CXCR3^–^CCR6^+^) were lower compared with healthy controls (Fig. 4G and H). Interestingly, the frequencies of cT_FR_ increased in the patient compared with healthy controls (Fig. 4I and J). These results revealed abnormal T-cell subtypes in STIM1 deficiency, characterized by increased T_H_1- and T_H_2-cell-like responses.

### Rapamycin Therapy Controls Lymphoproliferation and Reverses Lymphocyte Responses Caused by STIM1^Ex2del^

**Fig. 5:**
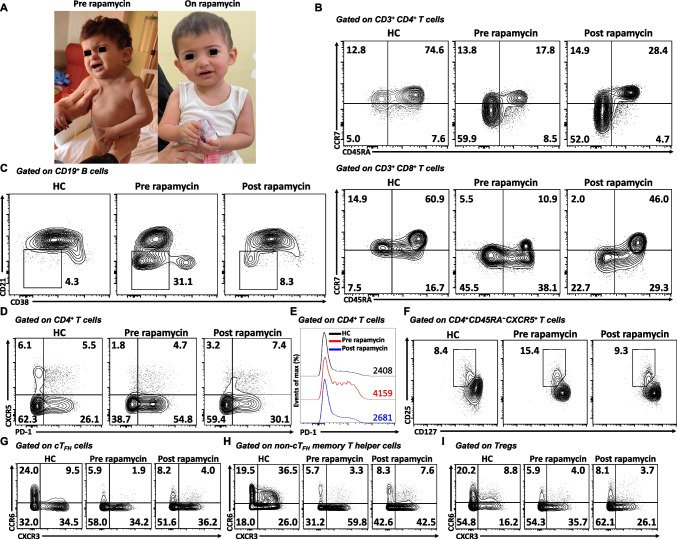
Rapamycin therapy controls lymphoproliferation and reverses lymphocyte responses caused by STIM1^Ex2del^. (**A**) Response of patient to rapamycin therapy. (**B**) Representative plots of T-cell subtypes of age-matched healthy control and pre- and post-rapamycin patient samples. Plots show CD4^+^ and CD8^+^ T cells that are naïve (CCR7^+^CD45RA^+^), central memory (CCR7^+^CD45RA^−^), effector memory (CCR7^−^CD45RA^−^), and exhausted (CCR7^−^CD45RA^+^). (**C**) Representative plots of CD21^low^CD38^low^ activated B cells of age-matched healthy control and pre- and post-rapamycin patient samples. (**D**) Representative plots of cT_FH_ cells of the age-matched healthy control and pre- and post-rapamycin patient samples. (**E**) Representative histograms of PD-1 expression in healthy control and pre- and post-rapamycin patient samples. (**F**) Representative plots of cT_FR_ cells of healthy control and pre- and post-rapamycin patient samples. (**G**) Representative plots of subtypes of the cT_FH_ cells of healthy control and pre- and post-rapamycin patient samples. (**H**) Representative plots of subtypes of the non-cT_FH_ memory T cells in the healthy control and pre- and post-rapamycin patient’s samples. (**I**) Representative plots of subtypes of Treg cells of healthy control and pre- and post-rapamycin patient’s samples. Pt: patients, HC: healthy controls, MFI: mean fluorescence intensity, cT_FH_: circulating T follicular helper cell, PD-1: programmed cell death protein 1, Treg: regulatory T cells, cT_FR_: circulating T follicular regulatory cell

Since the patient displayed an ALPS-like phenotype, we initiated oral rapamycin (1.3 mg/m^2^/once daily) to control the lymphoproliferation. After one month of the therapy (plasma rapamycin level: 8.3 ng/mL, therapeutic reference range: 5 – 15 ng/mL), the enlarged lymphadenopathies started to reduce in size and resolved within 3 months of treatment (Fig. 5A). Her blood leukocyte, lymphocyte, and eosinophil numbers and serum IgE levels all normalized within 3 months of treatment; however, non-immune defects, including myopathy and enamel hypoplasia had remained unchanged (Table 1). After 20 months of rapamycin therapy, she continues to do well without recurrence of lymphadenopathy. Rapamycin therapy was not associated with drug-related adverse effects or infections. At 3 years of age, she was transplanted from a full HLA-matched mother and is currently in the 2^nd^ month of the post-HSCT period.

**Fig. 6:**
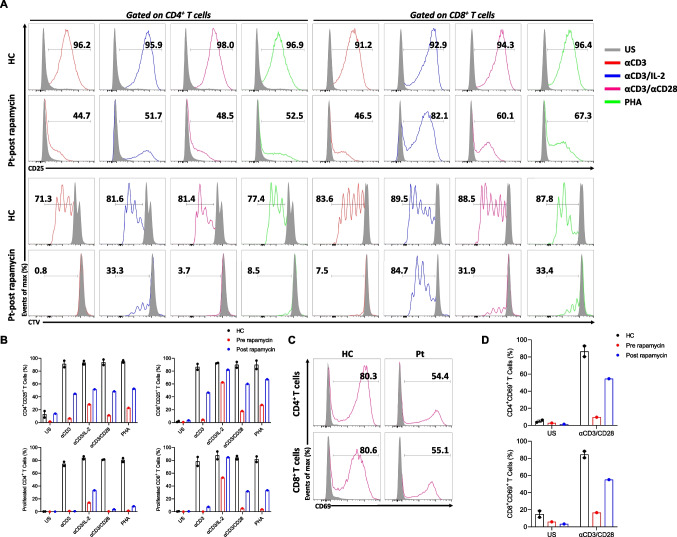
Rapamycin therapy partially restores T cell proliferation, CD25, and CD69 expressions in STIM1^Ex2del^ deficiency. (**A**) Representative flow cytometric analysis of percentages of CD25 (upper panel) and proliferation (lower panel) in CD4^+^ and CD8^+^ T cells of healthy control and patient post-rapamycin with unstimulated condition (grey) and stimulated (αCD3 (1 μg/mL, red line), αCD3/IL-2 (1 μg/mL and 100 U/mL, blue line), αCD3/CD28 (1 μg/mL each, purple line), and PHA (10 μg/mL, green line)) conditions for 72 h. (**B**) The percentages of CD25 upregulation and proliferation in CD4^+^ and CD8^+^ T cells of healthy controls and patient pre- and post-rapamycin with unstimulated and stimulated (αCD3, αCD3/IL-2, αCD3/CD28, and PHA) conditions. (**C**) Representative flow cytometric analysis of percentages of CD69 in CD4^+^ and CD8^+^ T cells of healthy control and patient post-rapamycin with stimulated (αCD3/CD28) condition. (**D**) The percentages of CD69 upregulation in CD4^+^ and CD8^+^ T cells of healthy controls and patient pre- and post-rapamycin with unstimulated and stimulated (αCD3/CD28) conditions. Pt: patient, HC: healthy controls, US: unstimulated, αCD3: anti-CD3, αCD3/CD28: anti-CD3/CD28, PHA: phytohemagglutinin, CTV: cellTrace Violet

Immunological studies were performed after 18 months of rapamycin. There was a marked restoration in the frequency of naïve T cells and decreased percentage of CD21^low^ CD38^low^ B cells (Fig. 5B and C). The percentage of cT_FH_ cells increased, and PD1 expression on CD4^+^ T cells declined with rapamycin (Fig. 5D and E). We further observed a decreased percentage of cT_FR_ cells (Fig. 5F). Together with the reversion of total cT_FH_ cells, we observed a slight reduction in the percentage of T_H_2-cell-like phenotype in this population (Fig. 5G). Rapamycin also reduced T_H_1-cell-like percentages in non-cT_FH_ memory T-helper and Treg cells (Fig. 5H and I). Similar changes were also detected in absolute numbers of these populations when compared with age-matched healthy controls (Fig.S3). The treatment partially restored the upregulation of activation markers and the proliferative capacity of T cells, particularly in CD8^+^ T cells, in response to stimulation with αCD3/IL-2, αCD3/CD28, and PHA (Fig. 6A and D). These results indicate that treatment with the mTOR inhibitor rapamycin improved the clinical and immunological findings associated with STIM1^Ex2del^ mutation.

## Discussion

This study describes a novel *STIM1* mutation in a child with severe lymphoproliferation, recurrent infections, myopathy, iris hypoplasia, and enamel hypoplasia. Lymphoproliferation was associated with severe T-cell infiltration without malignant transformation. The mutation resulted in complete loss of protein expression, accompanied by a lack of Ca^+2^ influx, defective T-cell activation, proliferation, and cytokine production. Furthermore, this mutation was related to increased eosinophil numbers and serum IgE levels conveyed by abnormal T_H_2 responses in T-cell subpopulations. Interestingly, the patient’s T cells showcased dysregulated T_FH_/T_FR_ responses that can contribute to the lymphoproliferative process. The efficacy of rapamycin to reverse T-cell responses and improve disease severity suggests aberrant mTOR signaling as a key pathomechanism of STIM1 deficiency.

Mutations within the *STIM1* gene precipitate either a complete loss or diminished protein levels, resulting in CID concomitant with ectodermal dysplasia and non-progressive muscular hypotonia [4, 9]. Patients generally suffer from severe recurrent life-threatening infections within the first year of age. Most patients are vulnerable to severe bacterial (Gram-positive and Gram-negative bacteria, *Mycobacterium bovis*), viral (typically with herpes virus infections, including those with CMV, EBV, and varicella-zoster virus), and fungal infections (*Pneumocystis jirovecii*, *Candida albicans*, or *Aspergillus fumigatus*) [3, 4]. They display autoimmunity mainly as immune thrombocytopenia and hemolytic anemia [3, 30]. Furthermore, this disease has distinctive non-immune clinical findings characterized by anhydrosis, iris hypoplasia, and myopathy. The severity of myopathy can vary, and in more severe cases, individuals may require a wheelchair for mobility [14]. Overall, although these unique findings enable faster diagnosis, variable and less prominent features were described in the literature (detailed clinical features of previously reported patients (P1-P17) are presented in Table S2). Accordingly, four previously described patients (P4, P7, P10, P11) showed no muscular problems. P7, P15, and P16 presented mild immunodeficiency characterized by recurrent respiratory infections, while others showed severe CID [13, 17].

The most prominent finding in our case was the drastic enlargement of multiple lymph nodes. This presentation might be confused with ALPS. Of the reported patients, lymphoproliferation was observed in seven, including our case (41%). However, only limited pathological examinations were conducted (P4, P5, and P17). In more detail, P4 showed lymphadenopathy related to Kaposi Sarcoma, and a skin biopsy revealed spindle cells and positive staining for HHV-8 [11]. P5 had destructive EBV-positive lymphoproliferation, and biopsies showed lymphocytic infiltration, including mainly CD3^+^ T cells and some CD20^+^ B cells. Focal accumulation of EBV-encoded small RNAs ( +) B cells was also noted. This patient responded well to the rituximab treatment [12]. Lastly, in P17, a liver biopsy was performed due to persistent hepatomegaly but did not reveal a specific diagnosis [18]. In our case, a biopsy from lymph nodes revealed lymphocytic infiltration consisting predominantly of T cells, leading to invasion and destruction of the typical nodal structure. Intriguingly, treatment with rapamycin yielded notable success in reducing lymphoproliferation, underscoring the significance of characterizing the cellular composition within the infiltrated tissue to guide treatment selection for enhanced symptom control.

Notably, the response of lymphoproliferation to rapamycin sheds light on the role of mTOR in SOCE channelopathies [31]. Rapamycin serves to expand and preserve the function of Tregs while impeding effector T cell proliferation. These actions highlight the diverse actions of mTOR signaling and their contribution to controlling immune regulation, possibly through influencing metabolic processes in Treg cells [32, 33]. A similar regulatory mechanism could be implicated in SOCE channelopathies, given the pivotal role of Ca^2+^ influx in activating TCR-induced calcineurin–NFAT and AKT-mTOR signaling pathways. These pathways, in turn, orchestrate the differentiation and proliferation of effector T cells by modulating transcriptional programs that establish phenotypic characteristics and ensure requisite metabolic adaptation [31]. Further investigations are warranted to provide a comprehensive understanding of how mTOR inhibition enhances the functional capacity of SOCE-negative effector T cells and Treg cells and to what extent improvements are due to metabolic reprogramming.

Insufficient development and functioning of Treg cells in SOCE channelopathies leads to autoimmunity and lymphoproliferative symptoms [2, 4, 9, 12]. Stim1/Stim2-deficient mice have demonstrated reduced Treg cells, potentially stemming from reduced NFAT activation, resulting in diminished *FOXP3* gene expression [4]. Interestingly, studies involving mice lacking Stim1 and Stim2 in mature Treg cells exhibited normal or even elevated FOXP3^+^ Tregs in their thymus and secondary lymphoid organs compared to control littermates. This underscores that the deletion of STIM1 and STIM2 in mature Tregs does not impede the maintenance of Treg cells. However, these Tregs demonstrated impaired immunosuppressive function and differentiation into T_FR_ and tissue-resident Treg cells [34]. Reduced peripheral blood Treg percentages were also reported in previous STIM1-deficient patients [5, 12]. In our patient, upon stimulation, we observed low percentages of Treg cells, accompanied by decreased expression of CD25, FOXP3, and CTLA4. This finding elucidates the essential role of STIM1 in maintaining the proper functionality of Treg cells and probably in controlling lymphoproliferation. Notably, STIM1 is known to regulate FOXP3 expression within CD4^+^ T cells and their differentiation into inducible Treg cells, which serve as gatekeepers of T-cell activation [4, 35]. Hence, Stim1-deficient mouse models have been shown to exhibit T-cell lymphocytosis and hyperinflammation during chronic infection with *Mycobacterium tuberculosis* [35]*.* Collectively, these findings emphasize the intricate relationship between SOCE channelopathy and Treg cells.

Mice deficient in both Stim1 and Stim2 in T cells develop a lymphoproliferative disorder with markedly increased number of splenic T_FH_ cells and diminished T_FR_ cells in secondary lymphoid organs. The imbalance between cell populations may be attributed to defective IL-2 production, leading to increased T_FH_ development [27, 28]. On the other hand, Vaeth et al. demonstrated imbalanced differentiation between T_FH_ and T_FR_ cells in aging Stim1/Stim2-deficient mice in secondary lymphoid organs. The disruption in T_FR_ cell differentiation was more pronounced than in T_FH_ cells. This disparity renders residual T_FH_ cells uncontrolled for the expansion of germinal centers and enhanced production of autoantibodies. Interestingly, in the same model, when these mice were exposed to infections, pathogen-specific T_FH_ and T_FR_ cells were reduced, resulting in defective germinal center reactions. These results implicate SOCE in governing the differentiation of T_FH_ and T_FR_ cells, possibly through NFAT-mediated expression of IRF4, BATF, and BCL-6 [29]. Similarly, our patient showed lower cT_FH_ but with an increased activation marker of PD-1. The defective SOCE would lead to unopposed activation in T_FH_ cells, potentially contributing to our patient’s lymphoproliferation.

It is well-demonstrated that STIM1 plays a critical role in regulating FAS ligand expression through the transcription factor of NFAT [36]. Mouse models with T cell-specific Stim1 deletion exhibited impaired apoptosis and activation-induced cell death pathways, which led to T-cell mediated hyperinflammation [35]. The impaired activation-induced cell death was also discerned in the human STIM1 deficiency [14]. This regulatory facet of STIM1 in determining T-cell survival provides another plausible mechanism for the lymphoproliferative symptoms evident in our patient.

In our case, rapamycin treatment increased cT_FH_ with reduced PD1 expression, and this reversion was concurrent with decreased cT_FR_, which was high at baseline. These data suggest a balanced, reciprocal alteration between these cell populations. The T_FR_ cells have never been tested before in human ORAI1- or STIM1-deficient cells; therefore, the changes in T_FH_ and T_FR_ cells may not be universal among the species as demonstrated to be reduced in mouse models. Another intriguing observation in human ORAI and STIM1 deficiencies is that normal levels of Treg cells with preserved suppressive function. These contrast what has been observed in Stim1/Stim2-deficient mice [12, 14–16]. These discrepancies warrant further investigations into how SOCE regulates Treg and T_FR_ numbers and their functions in human cells.

Our patient also exhibited eosinophilia and elevated IgE levels, which indicates a similar potential underpinning mechanism proposed by Oh-hara et al. in the Stim1/Stim2-double knockout mouse model. Their study unveiled the activation of the NFAT2 transcription factor required for T_FH_ development without Ca^+2^ entry, looping into the augmented IL-4 secretion from expanded T_FH_ cell populations. This perturbation increased class switching toward IgE and the induction of eosinophilia [28]. In concordance with these findings, eosinophilia and high IgE were observed in two other reported patients [15, 16]. These may be associated with unrestrained T_FH_ cell activation, which exhibited a T_H_2-like phenotype. Remarkably, rapamycin treatment effectively regulated this aberration, suggesting a compelling avenue for therapeutic intervention in such patients.

The previously described patients displayed normal frequencies of CD3^+^ T, CD19^+^ B, and CD16^+^CD56^+^ NK cells. Nevertheless, detailed immunophenotyping showed various immunological abnormalities. These include decreased naïve T cells, increased memory T cells, reduced B-cell subtypes, Treg, and invariant NKT cells [9, 16]. In our patient, reduced naïve CD4^+^ and CD8^+^ T cells and increased memory T cells supported the observed dysregulated phenotype in reported patients. Additionally, impaired T-cell proliferation is usually observed in STIM1 deficiency; however, this defect can be rescued by extensive costimulation by IL-2 and IL-7 cytokines by bypassing the TCR and SOCE dependency of T-cell proliferation [18]. On the other hand, studies have illustrated that CD8^+^ T cells, when stimulated ex vivo, often exhibit increased proliferation following exposure to rapamycin in vitro under TCR and CD28 costimulation [37, 38]. This supports the notion that the inhibitory effect of rapamycin on proliferation is predominantly observed when T cells receive TCR stimulation without concurrent costimulatory signals or IL-2 receptor signaling [39]. Nonetheless, beyond stimulatory conditions, rapamycin induces anergy in proliferated T cells even in the presence of costimulation, representing a final effector output [40]. In our patient, the beneficial effects of rapamycin and IL-2 may serve as determining factors for increased proliferation, particularly in CD8^+^ T cells.

The clinical findings and immunological changes would be more overt in patients with severe null mutations (nonsense and deletion) [5, 18]. The described immunological findings in these patients with severe mutations would be more consistent with mice lacking Stim1/Stim2 proteins. On the other hand, missense mutations can have less profound clinical and immunological phenotypes by preserving functional STIM1 protein to some extent [12–15]. However, due to the few reported patients, an accurate genotype–phenotype assessment remains challenging since patients with mild immunological phenotypes are reported despite complete loss of STIM1 protein [15–17].

Another interesting observation in our patients is the elevated CD21^low^ B cells. This population has increased in common variable immune deficiency, presenting autoimmunity and splenomegaly [41]. Notably, subsets of CD21^low^ B cells are characterized by the expression of T_H_1 transcription factor T-bet, contributing to the development of autoimmunity in inborn errors of immunity patients [42]. Elevated CD21^low^ B cells in our patients could be an alternative pathway that facilitates autoimmunity in this disorder in addition to, or apart from defective Treg cell function.

In conclusion, intracellular calcium dynamics profoundly impact lymphocyte biology and, thus, immune homeostasis. Disruption of calcium entry into the cell due to *STIM1* mutations causes various perturbations in lymphocyte functioning, resulting in severe immune dysregulation. Targeting the mTOR pathway with rapamycin might elicit immune modulatory effects sufficient to control the immune dysregulation in CRAC channelopathies. Rapamycin did not expose our patient to additional side effects during the study. However, it is imperative to exercise caution when employing immunosuppressants in individuals with immunodeficiency. Further work to substantiate these claims and to investigate the discrepancy between human and mouse STIM1 deficiencies is warranted to understand the precise role of SOCE in human subjects.

### Supplementary Information

Below is the link to the electronic supplementary material.

Supplementary file1 (PDF 115 KB)

Supplementary file2 (PDF 530 KB)

Supplementary file3 (PDF 71 KB)

Supplementary file4 (XLSX 18 KB)

Supplementary file5 (DOCX 30 KB)

Supplementary file6 (DOCX 24 KB)

## Data Availability

The data generated during the study are included in this published article.
