# Supplementary figures and images for: Rapamycin Controls Lymphoproliferation and Reverses T-Cell Responses in a Patient with a Novel STIM1 Loss-of-Function Deletion
Source: J Clin Immunol. 2024 Apr 5;44(4):94. doi: 10.1007/s10875-024-01682-0 (PMC10997552; doi:10.1007/s10875-024-01682-0)

Figure S1

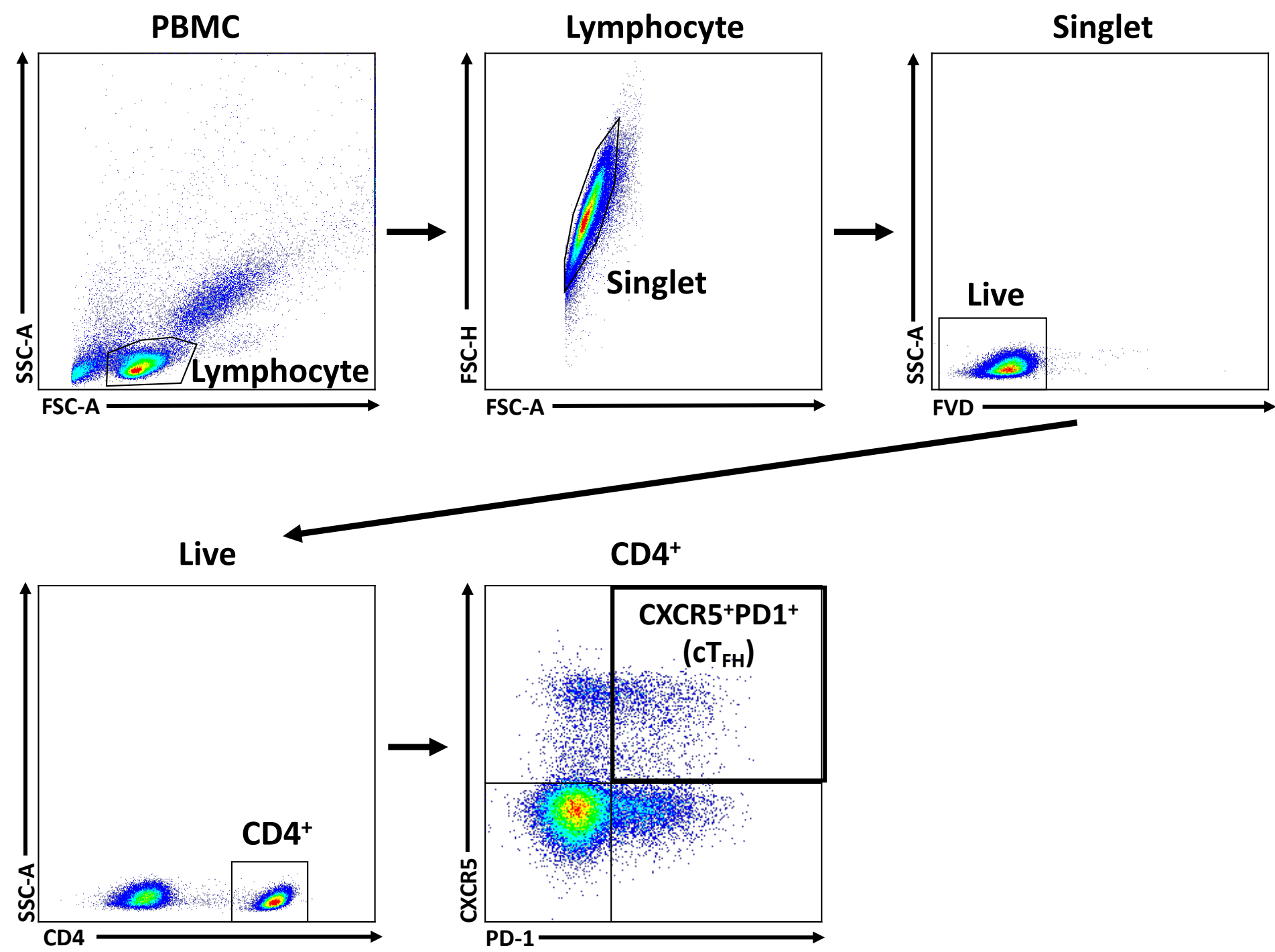

Supplement: Supplementary file 1 — Supplementary file1 (PDF 115 KB) [file 10875_2024_1682_MOESM1_ESM.pdf]

Figure S2

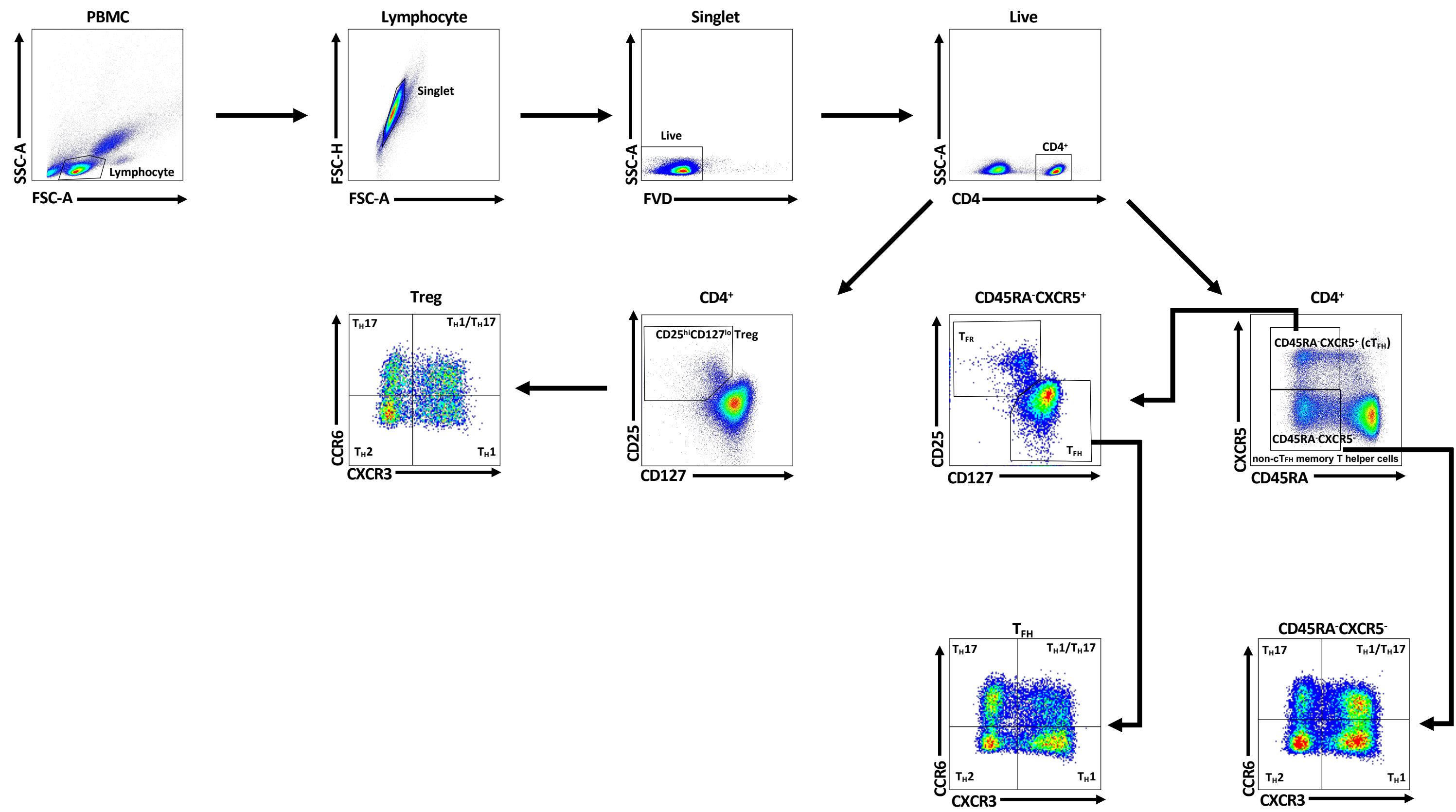

Supplement: Supplementary file 2 — Supplementary file2 (PDF 530 KB) [file 10875_2024_1682_MOESM2_ESM.pdf]

Figure S3

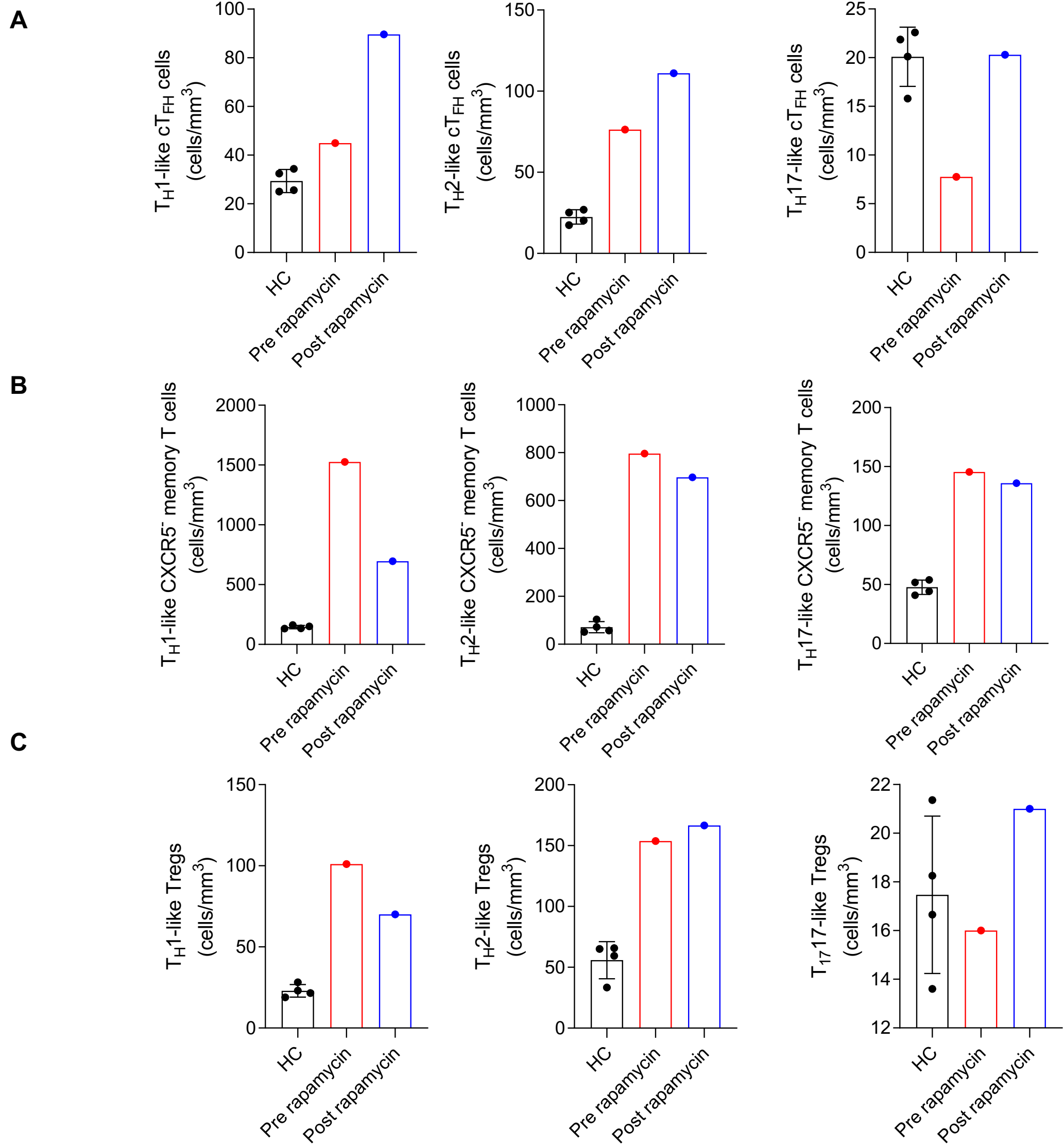

Supplement: Supplementary file 3 — Supplementary file3 (PDF 71 KB) [file 10875_2024_1682_MOESM3_ESM.pdf]
