# Supplementary material for: Rapamycin Controls Lymphoproliferation and Reverses T-Cell Responses in a Patient with a Novel STIM1 Loss-of-Function Deletion
Source: J Clin Immunol. 2024 Apr 5;44(4):94. doi: 10.1007/s10875-024-01682-0 (PMC10997552; doi:10.1007/s10875-024-01682-0)
Supplement: Supplementary file 5 — Supplementary file5 (DOCX 30 KB) [file 10875_2024_1682_MOESM5_ESM.docx]

| Patient | This report | P1 | P2 | P3 | P4 | P5 | P6 | P7 | P8 | P9 | P10 | P11 | P12 | P13 | P14 | P15 | P16 | P17 |
| --- | --- | --- | --- | --- | --- | --- | --- | --- | --- | --- | --- | --- | --- | --- | --- | --- | --- | --- |
| Mutation (NM_003156) | Exon 2 deletion | E136X | Unknown | E136X | 1538-1G>A | R429C | R429C | R426C | P165Q | P165Q | L74P | L74P | L374P* | S160fs* | T273fs | F229Lfs*12 | F229Lfs*12 | L374P* |
| Reference | - | Picard et al. | Picard et al. | Picard et al. | Byun et al. | Fuchs et al. | Fuchs et al. | Wang et al. | Schaballie et al. | Schaballie et al. | Parry et al. | Parry et al. | Vaeth et al. | Rice et al. | Derakhshan et al. | Salvi et al. | Salvi et al. | Kahlfuss et al. |
| Age of Onset | 16 months | 7 months | <18 months | 2 months | 24 months | 10 weeks | <21 months | 6 years | 6 months | 3 months | 18 years | 11 years | First weeks  of life | 6 months | Early Infancy | 3 years | 8 years | 2 weeks |
| Immunodeficiency | + | + | + | + | + | + | + | - | + | + | - | - | + | + (mild) | + | + (mild) | + (mild) | + |
| Dysmorphic face | - | NR | NR | NR | NR | NR | NR | NR | NR | NR | NR | NR | NR | NR | NR | + | + | NR |
| AIHA | - | + | + | - | + | + | + | - | - | - | - | - | - | - | + | - | - | - |
| ITP | - | + | + | + | - | + | + | - | - | - | - | + | - | - | + | - | - | - |
| Lymphadenopathy | + | + | + | - | + | + | - | - | + | - | - | - | - | - | + | - | - | + |
| Hepatosplenomegaly | + | + | + | - | + | - | - | - | + | - | - | - | - | - | - | - | - | + |
| Myopathy | + | + | + | + | - | + | + | - | + | + | - | - | + | + | + | + | + | + |
| Eosinophilia / IgE level | High / High | NR / NR | NR / NR | NR / NR | NR / NR | Eosinophilic inflammation / Normal | NR / Normal | NR / NR | NR / Normal | NR / NR | NR / Normal | NR / High | Normal / Normal | NR / High | NR / Normal | NR / NR | NR / NR | Normal / Normal |
| Eye Findings | Mydriasis/ Partial iris hypoplasia/ Non-reactive pupil | Mydriasis/  Partial iris hypoplasia | Mydriasis/ Partial iris hypoplasia | Mydriasis/  Partial iris hypoplasia | - | Mydriasis | Mydriasis | - | - | - | - | - | Partial iris hypoplasia/ Absence of sphincter muscle | Mydriasis | Partial aniridia/Non-reactive pupil | - | - | Mydriasis/ Partial Aniridia |
| Anhydrosis | + | NR | NR | NR | NR | + | + | - | + | + | + | + | + | + | Hypohydrosis | - | - | + |
| Enamel Defect | + | + | + | + | NR | + | + | + | + | + | + | + | + | + | - | + | + | - |
| Sparse hair | - | NR | NR | NR | NR | NR | NR | NR | NR | NR | NR | NR | + | NR | NR | NR | NR | + |
| Skin Involvement | Xerosis | - | - | - | Eczema, Kaposi sarcoma | Eczema | Eczema | - | Psoriasis | Chronic dermatitis | Eczema | - | - | Eczema Ichythosis | - | Hyperlaxity | Hyperlaxity | Seborrheic dermatitis |
| Nail Involvement | - | - | - | - | - | Nail Dysplasia | - | + | - | Brittle Nail | - | - | Nail Dystrophy | Nail Dystrophy | Nail Dysplasia | - | - | - |
| GI Involvement | - | - | - | - | - | Colitis | - | - | Colitis | - | - | - | - | - | - | - | - | Crohn’s disease |
| Nephrotic Syndrome | - | - | + | - | - | - | - | - | - | - | - | - | - | - | + (FSGS) | - | - | - |
| EBV-related LPD/lymphoma | - | - | - | - | - | + (LPD) | - | - | - | - | - | - | + (Lymphoma-received anti-CD20) | - | - | - | - | - |
| Abnormal skelatal survey | - | NR | NR | NR | NR | NR | NR | NR | NR | NR | NR | NR | NR | NR | NR | Hypoplastic patellae | Hypoplastic patellae | Hyperextensibility |
| Vasculitis | - | - | + | - | - | - | - | - | - | - | - | - | - | - | + (CNS and lung) | - | - | - |
| Creatine Kinase | N | NR | NR | NR | NR | NR | NR | NR | NR | NR | N | N | NR | N | NR | NR | NR | NR |
| HSCT | - | + | - | + | - | + (Rejected at the first attempt) | - | - | - | - | - | - | - | - | - | - | - | - |
| Status | Alive | Deceased | Deceased | Alive | Deceased | Alive | Deceased | Alive | Alive | Alive | Alive | Alive | Deceased | Alive | Alive | Alive | Alive | Alive |
| Reason of Death | - | HSCT complication | Nephrotic syndrome, encephalitis, enterovirus | - | Pulmonary infection, disseminated Kaposi sarcoma | - | Sepsis | - | - | - | - | - | Respiratory failure (worsened by myopathy) | - | - | - | - | - |

**Table S2.** The clinical features and outcomes of our patient and previously reported STIM1-deficient patients

**Abbreviations**: AIHA: Autoimmune hemolytic anemia, CNS: Central nervous system, FSGS: Focal segmental glomerulosclerosis, GI: Gastrointestinal tract, HSCT: Hematopoietic stem cell transplantation, LPD: Lymphoproliferative disease, ITP: Immune thrombocytopenia, N: Normal, NR: Not-reported.
