# Supplementary material for: Rapamycin Controls Lymphoproliferation and Reverses T-Cell Responses in a Patient with a Novel STIM1 Loss-of-Function Deletion
Source: J Clin Immunol. 2024 Apr 5;44(4):94. doi: 10.1007/s10875-024-01682-0 (PMC10997552; doi:10.1007/s10875-024-01682-0)
Supplement: Supplementary file 6 — Supplementary file6 (DOCX 24 KB) [file 10875_2024_1682_MOESM6_ESM.docx]

**Rapamycin controls lymphoproliferation and reverses T-cell responses in a patient with a novel *STIM1* loss-of-function deletion**

**Karakus et al.**

**Supplementary Methods.**

**Flow cytometry and antibodies.** To determine detailed lymphocyte subsets, the following monoclonal antibodies (mAbs) were used: Fluorescein isothiocyanate (FITC)-conjugated CD3 (UCHT1, BC, FRA), Allophycocyanin (APC)-Alexa Fluor 700 (APC-A700) CD4 (13B8.2, BC), Krome Orange (KO) CD45 (J33, BC), Alexa Fluor 750 (APC-A750) CD45RA (2H4DH11LDB9, BC), Phycoerythrin (PE) CD197 (CCR7) (G043H7, BC), Pycoerythrin-Cyanin 7 (PC7) CD8 (SFCI21Thy2D3, BC), APC-A700 CD14 (RMO52, BC), PE CD16 (3G8, BC), Pycoerythrin-Cyanin 5.5 (PC5.5) CD56 (N901, BC), APC-A750 CD19 (J3-119, BC), Pacific Blue (PB) CD20 (B9E9, BC), PB CD21 (BL13, BC), PC5.5 CD38 (LS198-4-3 BC), PB CD31 (5.6E, BC), PC5.5 CD38 (LS198-4-3 BC), Phycoerythrin-Texas Red-x (ECD) CD45RO (UCHL1, BC), FITC IgD (IA6-2, BC), PB CD4 (RPA-T4, Biolegend), FITC CD45RA (HI100, Biolegend), PC5.5 CD25 (B1.49.9, BC), APC-A750 CD127 (R34.34, BC) PE CD183 (CXCR3) (G025H7, Biolegend), APC CD185 (CXCR5) (J252D4, BC), PC7 CD196 (CCR6) (B-R35, BC), PE CD279 (PD-1) (PD1.3, BC). For lymphocyte subset analysis, 100µL of whole blood was incubated with mAbs against surface markers for 20 mins in the dark at room temperature (RT). Red cells were lysed and washed before acquisition. For circulating T follicular helper cell (cT_FH_) and regulatory T (Treg) cell subtypes, total peripheral blood mononuclear cells (PBMCs) from both healthy controls and patients were stained using PB CD4 (RPA-T4, Biolegend), FITC CD45RA (HI100, Biolegend), PC5.5 CD25 (B1.49.9, BC), APC-A750 CD127 (R34.34, BC) PE CD183 (CXCR3) (G025H7, Biolegend), APC CD185 (CXCR5) (J252D4, BC), PC7 CD196 (CCR6) (B-R35, BC), PE CD279 (PD-1) (PD1.3, BC) for 20 minutes in the dark at RT. Cells were acquired by Navios EX cytometer (Beckman Coulter) and analyzed using FlowJo software (TreeStar, Ashland, Ore). CD25 upregulation and proliferation assay were performed by stimulation in anti-CD3 (OKT3, Biolegend) (1μg/ml), anti-CD3 (OKT3, Biolegend)/IL-2 (1 μg/mL, 100 U/mL), anti-CD3 (OKT3, Biolegend)/anti-CD28 (CD28.2, Biolegend) (1 μg/ml each), PHA (10 μg/ml) 96-well plates for 3 days, following isolated PBMCs labeling with CellTrace Violet (Thermo Fisher). After the stimulation, cells were stained with APC-A700 CD4 (13B8.2, BC), PC7 CD8 (SFCI21Thy2D3, BC) and PC5.5 CD25 (B1.49.9, BC). CD69 upregulation assay was performed by stimulation in anti-CD3 (OKT3, Biolegend)/anti-CD28 (CD28.2, Biolegend) (1 μg/ml each) 96-well plates for 24 hours. After the stimulation, cells were stained with APC-A700 CD4 (13B8.2, BC), PC7 CD8 (SFCI21Thy2D3, BC) and APC CD69 (FN50, Biolegend). Stained cells were acquired by Navios EX cytometer (Beckman Coulter) and analyzed by FlowJo software (TreeStar, Ashland, Ore).

For intracellular IL-4, IL-17A, IL-10, and IFN-γ detection, cell suspensions (1x10^6^ cells) were incubated with protein transport inhibitor containing monensin (BD Bioscience, USA), PMA (50 ng/mL) and ionomycin (1 μg/mL) for 6 h. After incubation, cells were fixed. For intracellular cytokine staining, fixed cell pellets were resuspended in a permeabilization reagent containing saponin (Thermo Fisher Scientific, USA), mixed with fluorescent-labeled cytokine-specific antibodies and antibodies against CD4, CD45RA, and CD45RO. After 45 mins of incubation, cells were washed twice and acquired by Navios EX cytometer (Beckman Coulter) and analyzed by FlowJo software (TreeStar, Ashland, Ore).

**Intracellular staining of FOXP3 and CTLA-4 proteins**

Intracellular FOXP3 and CTLA4 levels were determined in PBMCs without stimulation and after stimulation with anti-CD2, anti-CD3, and anti-CD28 T-cell expander dynabeads (Miltenyi Biotec, Bergisch Gladbach, Germany) at a ratio of 1 bead to 2 cells for 16 hours. After stimulation, Cells were stained with FITC CD3 (UCHT1, BC), APC-A700 CD4 (13B8.2, BC), PC5.5 CD25 (B1.49.9, BC) and ECD CD45RO (UCHL1, BC) for cell surface staining. After cell surface staining, cells were fixed and permeabilized (eBioscience FoxP3 staining kits, San Diego, Calif, USA). Permeabilized cells were stained for FOXP3 and CTLA-4 at room temperature for 60 minutes. Patient samples were analyzed with healthy control samples. All stained cells were acquired with a Navios EX cytometer (Beckman Coulter) and analyzed with FlowJo software (TreeStar, Ashland, Ore) and Kaluza Analysis Software (Version 2.1).

**Supplementary Figure and Table Legends**

**Figure S1.** The gating strategy of the T-cell subtypes.

**Figure S2.** The absolute numbers of T cell subsets in STIM1-deficient patient compared to age-matched healthy controls.

**Table S1.** The other rare variants observed in whole exome sequencing in STIM1-deficient patient.

**Table S2.** The clinical features and outcomes of our patient and previously reported STIM1-deficient patients.
